# Successful Aging and Frailty: A Systematic Review

**DOI:** 10.3390/geriatrics3040079

**Published:** 2018-11-15

**Authors:** Darryl Rolfson

**Affiliations:** Division of Geriatric Medicine, Department of Medicine, University of Alberta, Edmonton, AB T6G 2P4, Canada; darryl.rolfson@ualberta.ca; Tel.: +1-780-492-6233

**Keywords:** frailty, successful aging, multidimensionality, definitions, resilience, healthy aging, fitness, intrinsic capacity

## Abstract

The terms successful aging (SA) and frailty appear to have much in common, both in terms of overlapping constructs and common challenges with consensus and operationalization. The aim of this review is to summarize existing literature that defines that relationship. Primary and secondary source articles that used either term in the title or abstract were systematically reviewed for relevance to the study objective. Of 61 articles that met these criteria, 30 were secondary source, and of these four were highly relevant. Four of the remaining 31 original research articles were selected, and the prevalence of frailty and SA in populations with different characteristics were described and compared. The same model of frailty was used in all primary studies, but definitions for successful aging were heterogeneous. The prevalence of frailty ranged from 11.8% to 44.0% and that of SA ranged from 10.4% to 47.2%. The definitions used for each, especially the extent of multidimensionality, appeared to reflect the degree of overlap between SA and frailty. Whether frailty and SA are part of the same or different constructs, there is a pressing need for an ordered taxonomy to advance research that translates into clinical practice.

## 1. Introduction

It has been six decades since the term “successful aging” (SA) was first coined [1], and there is still poor consensus on how it should be defined and operationalized [2,3]. Likewise, after three decades since frailty was introduced as an important clinical construct [4], a single meaning and measure has not found broad acceptance. It can be argued that successful aging and frailty are different terms derived from similar and overlapping constructs. Thereby, both terms are mired in common challenges with definition and operationalization, resulting in similar impediments to progress in research and widespread application.

Why are the wheels still spinning? The demographic imperative of population aging has been long anticipated, and now with the arrival of a population swell of aging baby-boomers, it is imperative to create an ordered understanding and taxonomy for frailty, successful aging, and even other closely related terminology such as fitness, healthy aging, resilience, and intrinsic capacity. To illustrate the notion that SA and frailty may belong to a common paradigm, the following two examples are provided. One proposed continuum places fitness and frailty at opposite ends, allowing a description of aging attributes, and a discrimination of incremental change [5,6]. A slightly different model places successful aging and disability at opposite ends, describing frailty as a transition state [7]. There is a need to find a lexicon that involves a more natural use of in everyday language by older adults, health care professionals, researchers, and policy-makers in ways that both respect the dignity of every older adult, but also motivate research that translates into policy, products, and practice.

While there is a rich literature on the conceptual models of successful aging, it is more challenging to find operational definitions to match these models. The best known three-factor model of Rowe and Khan [8]—defined as freedom from disease and disability, high cognitive and physical functioning, and active engagement with life—was broadly adopted, and was appealing for clinical research. These criteria, which aimed to distinguish successful from usual and pathological aging, were adopted in 1984 by the MacArthur Network, supported by the National Research Council, to find consensus and advance research. However, as this work progressed, the criteria were criticized for being too narrow in scope, resulting in an ideal that is rarely achieved. In fact, Rowe and Kahn conceded the need for a revised model that captures social and subjective factors, and embraced a dialogue that would strengthen the model [9].

More contemporary models of SA, as detailed in an excellent comprehensive review [2], could include Selective Optimization with Compensation (SOC), Preventive and Corrective Proactivity (PCP), and several other objective, subjective, and cultural models that have been proposed over the past decade. SOC is grounded on a life-course view in which success is understood from a behavioral perspective; SA reflects outcomes that maximized gains and minimized losses. PCP defines successful aging as the extent to which older adults proactively adapt by calling upon internal coping resources and external social resources in the face of the real stressors of aging. In a 2006 comprehensive review of quantitative studies on SA, Depp and Jeste found no less than 29 different definitions used in large samples of community-dwelling older adults, with a predictably wide range of prevalence (0.4% to 95%). Recognizing these different viewpoints, they advocated for a broad definition that would encompass biopsychosocial aspects, and thereby be “acceptable to clinicians, researchers, and older adults alike” [10]. Phelan and Larson [11] conducted a literature review of definitions of SA and found seven key elements: life satisfaction, longevity, freedom from disability, mastery/growth, active engagement with life, high/independent functioning, and positive adaptation. They subsequently demonstrated a difference between the views of SA by researchers and by older adults themselves, justifying a definition that includes yet another viewpoint—the individual [12]. The meaning of SA outside of the clinical realm is clearly influenced by self-definition and cultural considerations [13].

The resemblance of the SA to the frailty story is uncanny, yet like twins separated at birth, it appears that the two topics have been nurtured by parallel research communities acting largely in isolation. The use of the term frailty rose in popularity from the mid-1980s, with proponents of both the narrower and the more expansive definitions from the start. During this time, efforts to harmonize the clinical construct of frailty with purported physiological underpinnings were ongoing [14], resulting in one model that defined a cycle of dysregulated energetics that could be linked to a recognizable clinical syndrome [15]. However, these efforts were balanced with compelling arguments that aging results in an accumulation of “deficits” that more comprehensively capture stochastic aspects of frailty, and allow its operationalization using a multidimensional set of variables [16]. This led to calls for a consensus definition and operationalization of frailty beginning at least as early as 2000 [17], with a lucid review of the various models, definitions, and criteria three years later [18]. Around that time, the notion that there were but two dominant operational models emerged: namely, the Cardiovascular Health Study (CHS)Frailty Phenotype model [19] and the Frailty Index (FI) [20,21]. After more than a decade, neither the CHS Phenotype measure nor the Frailty Index have been employed broadly in clinical care, with the exception of an electronic version of the FI [22]. 

In 2013, a Delphi process was used to discover the level of agreement by top experts drawn from different conceptual traditions regarding a comprehensive set of statements regarding frailty [23]. A high level of agreement was found on the idea that frailty is a multidimensional syndrome characterized by decreased reserve and diminished resistance to stressors. The group defined those dimensions as physical performance, nutritional status, mental health, and cognition. There was strong agreement that it is useful to define frailty in clinical settings to allow for prevention and treatment, but there was no agreement on what that operative definition or set of clinical or laboratory biomarkers should be. What has emerged in clinical settings is a set of categories of frailty measures that are being variously adopted depending on the particular needs in different research and clinical settings [24], and a need to tailor implementation accordingly [25]. With the closely related concepts of frailty and resilience still being viewed as a ‘work in progress’ [26], it is time to acknowledge all that has been accomplished and determine what now needs to be done to translate models and measures of frailty into meaningful applications. 

Thus, it would appear that the SA and frailty stories have much in common. First, the models seem to have nearly identical conceptual underpinnings in the continuum of aging from fitness to disability. Specifically, both have embraced biomedical explanations, life course views, functional performance, and multidimensional characteristics, both intrinsic and extrinsic. Second, in both cases, academic communities have created a spectrum of constructs from very narrow and biomedical to very broad, including functional and social factors. Third, there are similar strategies to operationalize both frailty and SA including biomarkers, physical phenotypes, performance-based measures such as gait speed, and multidimensional models that include both social aspects and subjective components. 

Fourth, both terms seem to stir up controversy, especially amongst those who find themselves being defined as part of the in-group or out-group. When successful aging is narrowly defined, the term communicates elitism and a sense of disempowerment for the majority of older adults who would be defined as unsuccessful by exclusion. Likewise, frailty is not a term by which older adults would choose to self-identify. However useful the term may be for clinicians and researchers, if it is understood as a stigmatizing condition in the public realm, then labeling may at best prevent implementation and at worst have unintended negative consequences [27,28]. 

To navigate a way forward, a common understanding and language for both SA and frailty would hasten research and streamline the implementation of the best strategies to optimize the experience of aging for each individual. Given the broadly anticipated research and clinical contexts in which SA and frailty may be of interest, it will be important to resist the urge to unnecessarily categorize these entities, and see them instead as variables, however defined. For example, frailty may be seen as an observable trait (physical phenotype), as a medical condition, as a geriatric syndrome, or as a state of exaggerated risk. The purpose of this review is to summarize current literature that explicitly examines the relationship between frailty and successful aging. 

## 2. Methods

All articles involving humans, between 1946 and August 2018, using both the terms “successful aging” (or “successful ageing”) and “frailty” in the title or abstract were identified using Medline. Secondary sources (review, expert recommendations, or other opinion-based articles) were considered separately from primary sources that provide new experimental results. The title and abstract of each article were reviewed in English to determine relevance for this review. More relevance was determined by the degree to which study objectives emphasized the relationship between frailty and successful aging, as detailed separately below for primary and secondary sources. Where the abstract suggested even possible relevance, an English translation of the full article was obtained for a more thorough review. Articles that were not filtered by Medline as “human”, but those found to be based on a human population were recovered to be considered for classification. 

To be classified as primary or original research on this topic, articles could employ either experimental or non-experimental design (quantitative, qualitative, or mixed methods), but were required to make the relationship between SA and frailty a clear study objective, and provide explicit criteria to operationalize both frailty and successful aging. Articles that described only methodology without providing results and articles that focused primarily on aging biomarkers were not included. Included articles were then rated for relevance to our study objective of the relationship between frailty and successful aging based on the inclusion of this in the article objectives. 

Articles that were not classified by Medline as a “review article” but found to be anything but original experimental research were considered to be a secondary source. For the purpose of this review, secondary sources were divided into (1) systematic reviews, (2) thorough literature reviews, (3) articles that offer consensus statements, expert meeting summaries, or policy recommendations, and (4) author opinions with light literature review. All were also rated for study relevance as high, medium, or low, as judged by the present author.

Explicit criteria for frailty was defined generously, given the broad strategies for its operationalization. Consistent with a classification used in a reviews on frailty in acute care [29] and in primary care [24], this could include a judgement-based measure, a physical performance-based measure, physical frailty (or phenotype), the Frailty Index, or a multidimensional measure. Likewise, in the absence of a criterion standard for successful aging, the use by study investigators of any operational definition of SA that resembled one of the aforementioned models was deemed to be adequate for this review. 

## 3. Results

### 3.1. Overview of Literature

The search resulted in 1734 articles that included the term successful aging (or ageing) and 9202 articles that included the term frailty in the title or abstract. Sixty-four articles included both terms, with 50 classified as human. However, upon closer reading, 12 of the 14 excluded articles were reclassified as human, and two articles reported on the same original research and were thus combined for this analysis. 

Of these 61 unique articles, 22 were classified by Medline as a review article, and confirmed upon closer reading as a secondary source. Another nine not so classified in Medline were also classified as secondary source articles after closer reading. After review of the 31 apparent secondary source articles, one was reclassified as an original article. Of the remaining 30 secondary source articles, four [30,31,32,33] were of high or medium relevance to this topic, and none of these were systematic reviews. Those that were deemed to be of low relevance did not directly address the relationship between the constructs of frailty and successful aging. 

Of the 31 original articles, only four [34,35,36,37] employed explicit criteria for both frailty and successful aging (See Table 1). None of these had a biomarker focus and all reported study results. All used a cross-sectional analytic design. The primary objective and actual findings in four studies was to establish an association between frailty and SA, while in one [38] the purpose was to use frailty and SA criteria to test for health inequality in a population. For frailty, all four used the Cardiovascular Health Study Phenotype criteria [19]. Each of the SA measures were a blend of functional independence, quality of life, cognition, and self-perceived health. None used standard SA criteria such as the three-factor model of Rowe and Kahn; however, all approximated these criteria using available measures as described below.

Briefly, the Cardiovascular Health Study (CHS) Phenotype measure [19] is a five-item scale that measures as present or absent, weight loss, slow walking speed, low levels of physical activity, subjective exhaustion, and weakness using explicit criteria. Pre-frail is defined as a score of 1 or 2, and Frail is indicated by a score of 3 or higher.

The Short Form 36 (SF-36) [39] is a 36-item generic measure of health status, divided into eight subscales, and presents the physical and mental status separately. In the Vitality 90+ study [40], six different models of successful aging were compared, all with different constellations of sub-components which were grouped under the Rowe and Kahn three-factor model. Specifically, “physical” includes diseases, vision and hearing, and physical functioning; “psychological” includes depression, living will, and self-rated health, and “social” includes meeting children and telephone use.

The Barthel Index [41] is a simple measure of independence for basic activities of daily living with lower scores representing more dependence on a 20-point scale. Finally, there were two cognitive performance scales. The Mini Examen Cognoscitivo (MEC) [42] is a Spanish version of the 30-item Min-Mental Status Exam, in which 30 is the best score and a score less than 24 is abnormal. The Short Portable Mental Status Questionnaire by Pfeiffer [43] is a 10-item scale with 0 (no errors) as the best score and cut-points of 3, 5, and 8 representing mild, moderate, and severe cognitive impairment, respectively.

### 3.2. Original Articles

In a cross-sectional survey of 903 older adults without cognitive impairment in Taiwan [34], Li et al. defined the SA group as those in the top tertile of a quality of life measure, the SF-36, an estimated 10.4% of the population. Here, the SF-36 was justified as the ideal SA measure based on its multidimensionality, with special emphasis on the physical and mental components. Frailty was measured using the CHS Phenotype model criteria. The prevalence of SA was 10.4% and, compared to those who were not successfully aging (NSA), SA was significantly associated with all eight remaining quality of life domains on the SF-36. Those with chronic diseases or other health-related problems had a lower prevalence of SA compared to those without. Those who were younger (≤70 years old), who able to visit others at will, and who lacked a history of falls, pain, and sleep disorders were more likely to be considered to be aging successfully. Frailty was present in 11.8% of the group, and was independently associated with SA. The proportion of SA with frailty and pre-frailty was 0.9% and 7.2%, respectively. 

Herr et al. assembled a cross-sectional cohort of 2350 retired people recruited between 2008 and 2010 as a part of the SIPAF study (“Système d’Information sur la Perte d’Autonomie Fonctionnelle de la personne âgée”) in France [35]. Those that consented (18.9% of eligible participants) were assessed by a comprehensive geriatric assessment (CGA) in home settings, allowing comparison by age group (70–79, 80–89, and 90+ years old) of the prevalence of health and function, successful aging, frailty, and disability. SA was defined using the Vitality 90+ study [40] as good or fair self-perceived health in the absence of dementia, sensory impairments disability affecting activities of daily living (ADLs), depression, and social isolation. Frailty was defined using the CHS Phenotype criteria. Disability was defined as the need for help with one ADL. These criteria were used to then define four groups (Disability, Frailty, Intermediate, and Successful Aging), which were then compared by age group and sex. There was notable heterogeneity in the categorical proportions amongst different age groups and between sexes. Moving from the youngest to the oldest, the proportion with frailty increased from 9.5% to 25.3%, while the proportion with SA decreased from 38.7% to 9.1%, a pattern seen in both sexes but most pronounced in women. While the prevalence of chronic diseases did not change, older age conferred an increase in prevalence of functional limitations, sensory impairment, cognitive impairment, poor mood, and frailty. The overall proportions for successful aging and frailty were 25.7% and 16.6%, respectively. Within the successful aging group, 2.3% were classified as frail.

Ferrer et al. constructed a cross-sectional view of a cohort in Spain from the Octabaix study and provided follow-up data at 5 years [36]. This included baseline CGA and other medical, functional, and cognitive data collected in community-dwelling adults, born in 1924 (age 85 at baseline), and followed prospectively. Frailty was defined using the CHS Phenotype criteria, and SA was an abbreviate measure based on the Rowe and Kahn model, considering functional independence (Barthel Index), normal cognition (MEC Test), and community dwelling. A total of 273 patients were assessed in the second year of the study, with a prevalence of SA of 47.2% and a prevalence of frailty of 20.5%. In the not successful aging (NSA) group, the prevalence of frailty was 34.7%, and the component factors of frailty with the strongest association were low physical activity and muscle weakness. Notably, within the successful aging group, 4.7% were frail and 55% were pre-frail. After 5 years, the mortality rate was high (42.1%), and survivors had significantly greater functional independence and fewer comorbidities [44].

In another Hispanic population, this time in Mexico, Carrazco-Pena et al. sought to determine the frequency of SA and its association with states of frailty. Their cohort of 400 was a cross-sectional assembly of people age >60 years who attended an ambulatory clinic over one year. They were assessed for medical status, sociodemographic characteristics, anthropometric measures, and frailty using the CHS Phenotype criteria. SA was defined as functional independence (Barthel ≥ 90) and normal cognition (Pfeiffer ≤ 2 errors), and NSA was similarly defined (Barthel ≤ 85 and Pfeiffer ≥ 3). The prevalence of SA was 40.4% and that of frailty was 44.0%. Compared to the SA group, the NSA group had significantly worse scores in ADLs, cognition, and frailty. Amongst those defined as SA, 17.9% were classified as frail and 52.4% were pre-frail. Frailty was significantly associated with functional dependence and cognitive impairment. The NSA group was significantly more likely to have a low physical activity status. 

As shown in Table 2, all four groups used the CHS Phenotype for frailty. As such, this definition of frailty did not capture multidimensional aspects of frailty such as cognition, mood, or other geriatric syndromes, nor did it emphasize the state of frailty (i.e., deficit accumulation) or its dynamic aspects in relation to stress. For SA, all of the used measures demonstrated high cognitive functioning, but none met all three factors originally suggested by Rowe and Kahn. Li et al. and Carrazco et al. defined SA as only high cognitive and physical functioning. Herr et al. based their SA definition on fair or good self-rated health in the absence of disability, dementia, depression, visual or hearing impairment, and social isolation. Notably, this definition may not conform to the three-factor model, but it does attempt to capture self-rated SA. Ferrer et al. came closest to the three-factor definition, lacking only a measure for high physical functioning. Unlike the frailty measures, the SA measures were heterogeneous, thus making comparisons challenging.

Table 3 compares the prevalence of SA and frailty in the populations. Differences in frailty prevalence may reflect actual differences in the population samples. However, Li et al. reported a prevalence of only 11.8% in a group of community-dwelling older adults who had no cognitive impairment. In contrast, Carrazco-Pena et al. reported a very high prevalence of 44.0% in a group of similar age, but a very different socioeconomic and health status. Both Herr et al. in Spain and Ferrer et al. in France describe less filtered population samples, though the latter group was defined by a common birth year (age 86 years), which might explain their higher frailty prevalence of 20.5% (vs. 16.6%).

Compared to frailty, there is an even wider difference in the prevalence of SA between the four studies, and this may reflect the additional heterogeneity in their measurement strategies. For example, on the basis of their selected community-based sample alone, a relatively high prevalence of SA would be anticipated in the Li et al. cohort, but in fact, it was the lowest at 10.4%, possibly reflecting their narrower definition of SA (top tertile in SF-36 on both physical and cognitive subscales). A similar (and very high) prevalence of SA in the Spanish (47.2%) and Mexican (40.4%) cohorts was unexpected given that, despite similarly liberal SA definitions, there were differences in population characteristics and frailty prevalence.

Herr et al. confirmed that age influences the prevalence of both SA and frailty. SA prevalence dropped from 38.7% in septuagenarians to 22.8% in octogenarians and 9.1% in nonagenarians. An inverse pattern was seen for frailty rising from 9.5% to 18.4% to 25.3%.

If frailty and SA were at opposite ends of a commonly defined construct, then the SA group should not include those classified as frail or even pre-frail. Indeed, this was the pattern in the Taiwanese SA subgroup, with only 0.9% classified as frail and 7.2% classified as pre-frail. By comparison, in the Spanish SA subgroup, 4.7% were frail and 55% were pre-frail. The largest overlap was seen in the Mexican SA subgroup, with 17.9% frail and 52.4% pre-frail.

### 3.3. Secondary Source Articles

Bourddel-Marchasson and Berrut commented in 2005 on the challenges with defining both SA and frailty [33]. Very old individuals who cope well in spite of emerging frailty, multi-morbidity, and disability would otherwise be classified as “usual” or “pathological” aging according to the three-factor model. However, from a person’s perspective, dignity in the midst of gathering frailty and apparent loss of autonomy can and should be maintained. Indeed, older adults who revise their expectations and persevere with new realities can be said to cope successfully. Comprehensive geriatric assessment might thereby have a role in assisting older adults to successfully navigate new challenges and implement tertiary prevention strategies.

Lowry in 2012 proposed that physical disability models of aging might be one way to harmonize definitions of SA and frailty [31]. Here, active life expectancy (i.e., disability-free) can be a candidate measure for successful aging. They find some justification in this view from the aforementioned review [10] of multiple definitions of SA, in which disability and physical functioning emerge as key components. The World Health Organization (WHO) provides a framework (International Classification of Functioning, Disability, and Health) in which disability is positioned at the intersection between environmental (extrinsic) and personal (intrinsic) factors. Lowry made the case that, given the “complex relationship between health, mobility, and participation”, disability may be the interface that allows a common language for frailty and SA. This also agrees with the multidimensional nature of both SA and frailty. The degree of disability is then a bidirectional and dynamic continuum upon which individuals may slide based on the interplay of intrinsic and extrinsic factors. These authors go on to propose gait speed as a “useful single-item screening tool”, reflecting this continuum.

In a lucid and introspective review in 2015, Friedman et al. inquired whether the strong leadership by academic geriatricians in addressing frailty and complexity has come at the cost of withdrawing its societal influence on healthy aging. Likewise, the success in defining frailty and understanding its processes could be a “framework for addressing healthy aging” [32]. This would require a standard definition of SA, one that is harmonious with that of frailty. Further, rather than using limited time resources in only providing care to the oldest, most complex, and functionally impaired, geriatricians could redirect some of their influence to the promotion of SA from a societal and systems standpoint.

Finally, in a 2015 review, one that most closely aligns with the object of this review, Cosco et al. asked whether SA and frailty are different paradigms or two ends of a shared continuum [30]. It is interesting to note that in both SA and in frailty, biomedical definitions found early support and were commonly adopted for instrumentation in research. Supporting a shared continuum, both the frailty and SA constructs include physiological aspects of aging and offer biomedical measures expressed as functional terms. Suggesting different paradigms, the state of frailty emphasizes the result of accumulated deficits, while SA places greater emphasis on psychosocial assets and fostering resilience. Simply put, the continuum view seems to be supported by similar biomedical underpinnings, while the different paradigm view is becoming more apparent as psychosocial elements start to be included. 

## 4. Discussion

There is a paucity of both primary and secondary source biomedical literature aimed at understanding the relationship between frailty and successful aging. Amongst four different populations that employed a similar frailty measure there were very different strategies for measuring successful aging, making comparisons and conclusions very challenging. Frailty prevalence ranged from 11.8% to 44.0%. Both differences between populations and strategies used to measure SA might explain the different reported prevalences of SA between 10.4% and 47.2%. This highlights the need to find consensus on the meaning and operationalization of both frailty and successful aging.

This review captured both primary and secondary source literature in Medline. However, other databases such as PsychInfo and CINAHL Plus were not employed, with the recognition that some of the more qualitative SA literature might missing in this analysis. Heterogeneity in prevalence between studies was not explored for statistical significance because differences were dramatic and explainable by differences in measures. Another important acknowledgement is that this systematic review was conducted by just one reviewer, and no risk of bias tool was applied. Study eligibility was explicit. However, the selection of studies was open to minor bias and the relevance of secondary source articles was determined by author judgement alone.

As posited by Cosco et al. [30], it is crucial to determine whether frailty and SA are different paradigms or part of a common continuum. By comparing these four studies, it is apparent that the proportion who are classified as both SA and frail, or even SA and pre-frail is quite variable. The largest overlap is seen with the Mexican cohort, in which 70.4% of SA were frail or pre-frail. The smallest was the Taiwanese cohort, in which 8.1% were so classified. Certainly, definitions of both SA and frailty that strictly define both entities as polar opposites will exclude the central zone in a continuum where there are meaningful multidimensional variables. In studies with less strict borders, where operational definitions approach or even overlap, we enter a murky area where frailty and SA co-exist in multidimensional terms, and in this sense, may find greater authenticity for both definitions. Further, with a higher and higher prevalence of individuals in the overlap zone between definitions, one might expect that the unique aspects of the polar definitions of frailty and SA give way to a single paradigm that could be operationalize on an ordinal scale. Examples here might include a single paradigm such as disability [31] or the recently emerging WHO model of Intrinsic Capacity [7]. As mentioned in the introduction, it has been proposed that frailty could be viewed as a transition stage between SA and disability [7], and this would only work if a very multidimensional definition of frailty was applied to adequately bridge these two multidimensional counterparts. As demonstrated in one hospitalized cohort using the Edmonton Frail Scale [49], multidimensional frailty measures have strong associations with the dimensions they are intended to represent.

However, even then, it would be very challenging for a unidimensional measure to account for complete aspects of its parent paradigm. For example, in a recent review it was proposed that frailty is nothing less than an expression of the complex systems that comprise the human condition, and that it requires inputs and solutions from a wide range of disciplines to be best understood [26]. By comparing frailty to the Golden Gate Bridge, Kuchel argued that frailty is simultaneously a state of exaggerated vulnerability (stochastic frailty), a syndrome with clinical expression (phenotypic frailty), and an entity that only be understood as a response to its external context (dynamic frailty).

Another explanation for overlap would be that frailty and SA are indeed different paradigms. It is true that compared to definitions of frailty in the frailty literature, definitions of SA in the SA literature have embraced psychosocial elements more extensively in recent years. In a scoping review [50], Carver et al. addressed non-biomedical factors in SA, finding common constructs including optimism, engagement, resilience, spirituality, self-efficacy, and gerotranscendence. Certainly, if frailty is defined as a narrow construct, for example as the CHS Phenotype, then SA could distinctively capture this full range of psychosocial and dynamic elements that are otherwise missing. SA models that emphasize the dynamic response of an individual—such as optimization through compensation, or proactive strategies to minimize impactful deficits—are not currently part of any frailty model.

An unknown author said that “success is going from failure to failure without losing your enthusiasm.” This seems to capture the paradox that lies at the heart of successful aging and frailty. Researchers who find security in narrow and more biomedically defined modes of SA and frailty stand on opposite sides of a bridge of multidimensionality that could bring meaning to both sides. As definitions for frailty and successful aging approach one another, research communities in both biomedical and social sciences will need to work together to discover what has until now been missing from both definitions. Frailty researchers need to better understand whether their frailty can be better understood by including contextual factors such as culture, individual factors such as self-identity, and dynamic factors such as selective optimization with compensation (SOC), or preventative and corrective proactivity (PCP). Likewise, by exploring the frailty story, successful aging researchers might explore how to better operationalize SA in ways that lend to translations research and clinical application, or understand it as a complex whole. Both communities could then advise whether we are working with one or two paradigms and, if it is one, then what terminology might help advance care and foster healthy aging communities.

## Figures and Tables

**Table 1:** Original research articles that employed explicit criteria for frailty and successful aging.

Authors	Size	Age	Setting	Frailty Measure	SA Measure
Li et al., 2014	903	65+	Community-dwellers in Taiwan	CHS Phenotype	SF-36 ^1^
Herr et al., 2016	2350	70+	General population in France	CHS Phenotype	Vitality 90+ ^2^
Ferrer et al., 2014/17	273	86	Primary care centers in Spain	CHS Phenotype	Barthel/MEC ^3^
Carrazco et al., 2018	400	60+	Hospital outpatients in Mexico	CHS Phenotype	Barthel/SPMSQ ^4^

^1^ The Short Form 36 (SF-36) [39] is a quality of life measure. Successful aging (SA) is defined as the top tertile in physical and mental subscales. ^2^ Derived from Vitality 90+ study [40] Model 3, which defines SA in terms of self-rated health and absence of dementia, special sensory impairments, disability in activities of daily living, depression, and social isolation. ^3^ Functional independence (Barthel [41]), normal cognition (Mini Examen Cognoscitivo [42]) and community dwelling. ^4^ Barthel Index and the Short Portable Mental Status Questionnaire (or Pfieffer test) [43] for cognition.

**Table 2:** Comparison of operationalization of successful aging measures by study.

Authors	Diseases	Disability	Cognitive	Physical	Life Engagement
Li et al., 2014	NR ^1^	NR	SF36 P ^2^	SF36 M ^2^	NR
Herr et al., 2016	SR ^3^	Katz ^4^	MMSE/GDS ^5^	NR	Social isolation
Ferrer et al., 2017	Charlson ^6^	Barthel ^7^	MEC ^8^	NR	Living in community
Carrazco et al., 2018	NR	NR	Pfeiffer ^9^	Barthel	NR

^1^ Not reported. ^2^ Short Form 36 Physical (P) and Mental (M) subscales [39]. Highest quintile for both. ^3^ Self-rated. ^4^ Katz Index [45] for independence in activities of daily living (ADLs). Interpretation not reported ^5^ Mini-Mental Status Exam ≤ 20 [46] and 15-item Geriatric Depression Scale > 5 [47]. ^6^ Charlson Comorbidity Index [48] and clinical history. ^7^ Barthel Index > 90 [41] for independence in basic activities of daily living. ^8^ Mini-examen cognoscitivo ≥ 24. Adaptation of the MMSE in Spanish [42]. ^9^ Short Portable Mental Status Questionnaire (Pfieffer) ≤ 2 [43] and Barthel Index ≥ 90.

**Table 3:** Comparison of reported prevalence of frailty and successful aging by study.

Authors	Age (Years)	SA (%)	Frail (%)	Pre-Frail (%)	Not Frail (%)
Li et al., 2014	65+	10.4	11.8	47.4	40.8
Herr et al., 2016	70+	25.7	16.6		
Herr et al., 2016	70–79	38.7	9.5		
Herr et al., 2016	80–89	22.8	18.4		
Herr et al., 2016	90+	9.1	25.3		
Ferrer et al., 2017	86	47.2	20.5	54.2	25.3
Carrazco et al., 2018	60+	40.4	44.0		
